# Cell therapy in end-stage liver disease: replace and remodel

**DOI:** 10.1186/s13287-023-03370-z

**Published:** 2023-05-25

**Authors:** Xin-Hao Hu, Lan Chen, Hao Wu, Yang-Bo Tang, Qiu-Min Zheng, Xu-Yong Wei, Qiang Wei, Qi Huang, Jian Chen, Xiao Xu

**Affiliations:** 1 The Fourth School of Clinical Medicine, Zhejiang Chinese Medical University, Hangzhou, 310053 China; 2 Key Laboratory of Integrated Oncology and Intelligent Medicine of Zhejiang Province, Department of Hepatobiliary and Pancreatic Surgery, Affiliated Hangzhou First People’s Hospital, Zhejiang University School of Medicine, Hangzhou, 310006 China; 3 Zhejiang Chinese Medical University, Hangzhou, 310053 China; 4 NHC Key Laboratory of Combined Multi-Organ Transplantation, Hangzhou, 310003 China; 5 Life Sciences Institute, Zhejiang University, Hangzhou, 310058 China

**Keywords:** Cell therapy, Stem cell, End-stage liver disease, Cell transplantation, Liver regeneration

## Abstract

Liver disease is prevalent worldwide. When it reaches the end stage, mortality rises to 50% or more. Although liver transplantation has emerged as the most efficient treatment for end-stage liver disease, its application has been limited by the scarcity of donor livers. The lack of acceptable donor organs implies that patients are at high risk while waiting for suitable livers. In this scenario, cell therapy has emerged as a promising treatment approach. Most of the time, transplanted cells can replace host hepatocytes and remodel the hepatic microenvironment. For instance, hepatocytes derived from donor livers or stem cells colonize and proliferate in the liver, can replace host hepatocytes, and restore liver function. Other cellular therapy candidates, such as macrophages and mesenchymal stem cells, can remodel the hepatic microenvironment, thereby repairing the damaged liver. In recent years, cell therapy has transitioned from animal research to early human studies. In this review, we will discuss cell therapy in end-stage liver disease treatment, especially focusing on various cell types utilized for cell transplantation, and elucidate the processes involved. Furthermore, we will also summarize the practical obstacles of cell therapy and offer potential solutions.

## Introduction

The liver, a vital organ for survival, is responsible for bile production, nutrient metabolism, toxin removal, blood purification, and inflammation [1]. The prevalence of liver diseases, such as non-alcoholic fatty liver disease (NAFLD), non-alcoholic steatohepatitis (NASH) and metabolic liver diseases, has increased and may ultimately progress to end-stage liver disease such as liver failure and liver cirrhosis [2, 3]. The only efficient treatment for these diseases is orthotopic liver transplantation (OLT); however, suitable organ donors are insufficient to meet clinical demand [4]. Due to the shortage of healthy donor livers, a wide gap exists between the number of patients on the waiting list and the number of organs available, and those waiting for organ donation have a high mortality rate [5, 6]. Despite the emergence of novel surgical transplantation procedures such as split liver transplantation, the problem of donor liver scarcity has not been satisfactorily addressed [7, 8]. Fortunately, cell therapy, an increasingly popular strategy for treating end-stage liver disease, can effectively address the shortage of donor livers and reduce the need for invasive surgical procedures.

Cell therapy involves using cells of various types to remodel or replace damaged organs or tissues. The transformed cells may be injected into the liver locally or intravenously to restore liver function or encourage liver regeneration [9]. The therapeutic effect of cell therapy has been extensively studied in animal models [10, 11]. The first study on cell therapy was conducted in 1976 by Najarian, who transplanted allogeneic hepatocytes into a rat model of congenital enzyme deficiency disease via the portal vein [12]. Later, Mito et al. attempted to transplant hepatocytes into the spleen, suggesting that the spleen may be used as an ectopic liver [13]. In 1992, researchers successfully restored the liver function of a patient with hepatic encephalopathy and severe ascites by transplanting hepatocytes into the spleen [14]. Subsequently, Strom et al. proved the viability of human hepatocyte trans-splenial artery transplantation in patients with end-stage liver disease, and the spleens of transplanted patients displayed a characteristic hepatic cord structure [15]. Of the five patients who received treatment, three made a full recovery and were successfully bridged to OLT, while in specific individuals with acute liver failure, cell therapy has also been effective in bridging to OLT [16]. Since then, hundreds of clinical hepatocyte transplants have been recorded worldwide.

Compared with OLT, cell therapy offers many benefits among surgical techniques. From an operational standpoint, cell therapy is more feasible than OLT or split liver transplantation because (1) multiple recipients can receive hepatocytes from a single donor; (2) the procedure is less invasive and simpler; (3) donor cells can be cryopreserved and accessed as needed; (4) the recipient liver is not removed and can continue functioning normally if the treatment is unsuccessful; and (5) the cost is lower [17]. While cell therapy shows great promise, some significant issues remain unresolved. Transplanted hepatocytes do not proliferate in patients with metabolic illnesses when the liver is undamaged and entirely healthy, such as Crigler–Najjar syndrome or familial hypercholesterolemia, because the liver does not need such proliferation during physiological processes. To allow transplanted cells to proliferate, the liver must be pretreated to boost the proliferative advantage of donor cells [18, 19]. Furthermore, immunological rejection of transplanted cells in the liver presents a significant obstacle.

Overall, cell therapy is an effective and promising approach for end-stage liver disease, and much research has been performed in this field. Before OLT, cell therapy can be used as a bridge treatment. However, it also has limitations in cell acquisition engraftment, proliferation and delivery. The solutions usually include cytokine stimulation, immune regulation, organoids, hyaluronan matrix, reprogramming media with antioxidants, intrasplenic cell infusion and peritoneal delivery. In this review, we summarize the latest progress in end-stage liver disease using cell therapy.

## Diverse cell sources and therapeutic sites for cell therapy

**Fig. 1:**
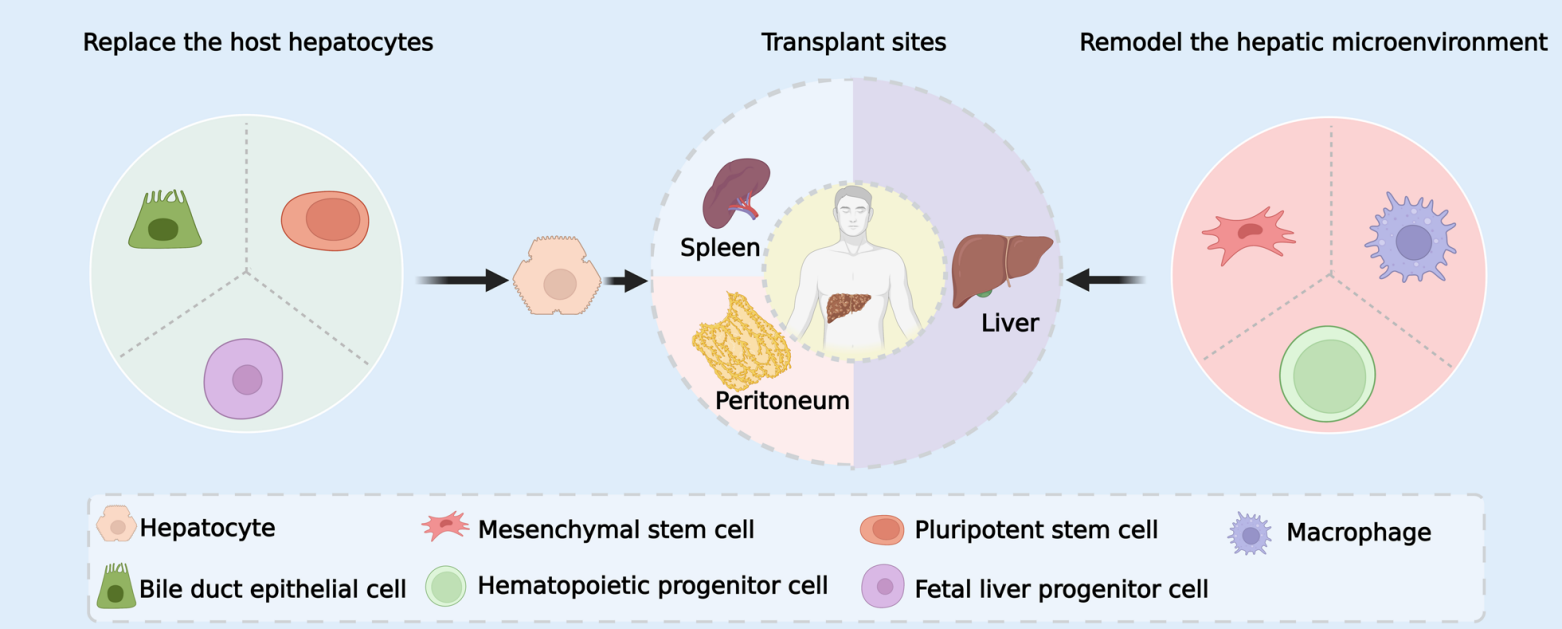
Cell sources and entry routes for cell therapy. Replacement and remodelling are the two primary purposes of cells deployed in cell therapy. Before cell transplantation, bile duct epithelial cells, pluripotent stem cells, and foetal liver progenitor cells must be differentiated into hepatocytes in vitro. Following transplantation, these cells proliferate within the liver and replace the host hepatocytes. Additionally, mesenchymal stem cells, macrophages, and hematopoietic progenitor cells can remodel the hepatic microenvironment. The liver, spleen, and peritoneum are all potential sites for cell transplantation

The hepatic lobule has a basic hexagonal shape, with the portal triad (the portal vein, bile duct, and hepatic artery) located at the lobule's periphery and the central vein in the middle. Hepatocytes, as primary parenchymal cells of the liver, perform most of the liver's physiological functions and maintain a certain liver-to-body ratio owing to the powerful regenerative capacity of the liver [20]. Thus, hepatocytes can be implanted into the liver to boost liver regeneration, meaning that transplanted hepatocytes multiplied and replaced host hepatocytes (Fig. 1). However, the acquisition and preservation of hepatocytes is a common problem. To obtain more hepatocytes, some teams gradually began to use stem cells or cells with stem cell properties that can transdifferentiate or differentiate into hepatocytes for cell transplantation (Fig. 1). Bile duct epithelial cells (BECs), for instance, can transdifferentiate into hepatocytes, which we believe is due to cell plasticity [21, 22]. Labelling BECs revealed that these cells can be converted to hepatocytes in the event of severe liver injury [21], implying that they are probably accessible as a source of transplantable cells. In recent studies, extrahepatic organoids derived from cholangiocytes have been demonstrated to preserve plasticity within the human biliary tree after transplantation and can repair human intrahepatic bile ducts [23]. This has piqued researchers' curiosity, and some have used human bile duct epithelial cells (hBECs) from discarded donor livers to rescue bile duct structure and function in a mouse model of biliary disease [24]. These studies showed that BECs may be an alternative cell source for cell transplantation. In further studies, pluripotent stem cells (PSCs) and foetal liver progenitor cells may differentiate in vitro into hepatocytes or hepatocyte-like cells for cell therapy, which will be described in detail later (Fig. 1).

In addition to replacing host hepatocytes and expanding within the liver, transplanted cells may remodel the hepatic microenvironment (Fig. 1). For example, transplanted cells can exert immunomodulatory, anti-inflammatory and antifibrotic effects on end-stage liver disease [25, 26]. Additionally, it has been shown that bone marrow transplanted cells, when abandoning transdifferentiation, may stimulate hepatocyte proliferation and restore liver function [27, 28]. In conclusion, transplanted cells can treat liver diseases by replacing host hepatocytes and remodelling the hepatic microenvironment (Fig. 1). Moreover, the feasibility of hepatocyte acquisition is also improved due to the plasticity of the cells [29].

In addition to the liver, some extrahepatic organs may serve as cell therapy sites. Currently, the more reliable transplantation sites are the spleen and peritoneum (Fig. 1). The transplanted cells may colonize these organs and perform the liver's functions [15, 30, 31]. In conclusion, the flexible cell therapy technique enables the selection of suitable transplanted cells and delivery organs according to the status of the specific patient.

### Replacing the damaged liver

#### Hepatocytes

Adult hepatocytes have been shown to expand in vivo, similar to haematopoietic stem cells [32]. Several successful genetic mouse models have been used to validate the use of cell transplantation to treat inborn errors of metabolism (IEM) of the human liver, including albumin-uPA transgenic mice, mice with alpha-1-antitrypsin deficiency, and those with fumaryl acetoacetate hydrolase deficiency (familial tyrosinemia) [33–35]. Within a few weeks, the transplanted cells effectively replaced most of the host hepatocytes in these immunodeficient mouse models, resulting in the formation of chimeric tissues. According to a case report in the New England Journal of Medicine, a 10-year-old girl with Crigler–Najjar syndrome type I and severe unconjugated hyperbilirubinemia was effectively treated by 7.5 × 10^9^ hepatocytes infused into the portal vein [36], and the therapeutic benefits for the patient persisted for up to 11 months and permitted a bridge to OLT [16]. Additionally, hepatocyte transplantation (HT) has been utilized to treat paediatric patients with liver metabolic abnormalities [37–39], and several investigations are being conducted to enhance the availability and safety of cells [40]. Even a small amount of host hepatocytes replaced by cell therapy produces significant clinical effects in treating IEM [41]. The efficacy of cell therapy has been shown in several illnesses, including phenylketonuria, Crigler–Najjar syndrome and propionic acidemia [42–44]. Most patients finally underwent OLT, and the greatly extended life before liver transplantation enhanced the likelihood that the patient would obtain a suitable donor liver. On the other hand, hepatocyte suspension can be safely administered into the portal vein, proving that cell therapy has a lower surgical risk than OLT. Strom et al. have shown the safety and therapeutic effectiveness of hepatocyte splenic artery infusion in patients with chronic end-stage liver disease [15]. However, the transplanted cells in some individuals do not dramatically proliferate but are progressively eliminated over several months. This occurs because the proliferation of transplanted cells in the liver necessitates a high proliferative advantage relative to the host cells and proper immunological microenvironment [45]. Therefore, a hypothesis can be put forth: halting the patient's current therapy may enable the patient's liver to have an environment beneficial to the engraftment of the transplanted cells.

Acute liver failure (ALF) is a rare acute disease with a high mortality rate [46]. An "indeterminate" aetiology is a reasonably frequent cause that often lacks a transparent causal element and affects people who have never had liver disease. OLT is the only effective treatment option [47]. Many studies have shown that primary hepatocyte transplantation may rescue animals from ALF and improve survival in a rat model where successful donor chimaerism occurs after transplantation [48–50]. The liver and spleen are the most reliable transplantation locations (Fig. 1) because their distinct vascular and extracellular matrix structures are ideal places for transplanted hepatocytes to stay and proliferate [50]. Alternatively, hepatocytes may be transplanted into the peritoneum [51]. Cell therapy is more suited for ALF patients with a typical liver structure. At several research centres, clinical hepatocyte transplantation studies have been performed. Strom et al. described five patients who underwent hepatocyte transplantation after perfusion of a mixture of 10^7^–10^9^ freshly isolated and cryopreserved hepatocytes through the splenic artery [52]. The four control individuals and all five test participants developed multisystem organ failure and grade IV hepatic encephalopathy. Among them, five participants were successfully bridged to OLT while maintaining normal cerebral perfusion and cardiac stability, whereas all four control subjects died within three days. At 20 months of follow-up, three of the five patients who successfully received liver transplantation were still alive and in good physical health. It remains challenging to use HT to provide consistent therapeutic benefits. After the transplanted cells are fully assimilated into the body, macrophages are activated and begin to phagocytose diseased cells and other debris. They also secrete transforming growth factor-β (TGF-β), which causes hepatocytes in the liver to undergo replicative senescence, which is then transmitted to the transplanted cells [53]. Smad2/3 is phosphorylated by the TGF-β signalling pathway, which activates downstream molecules such as extracellular matrix (ECM) genes and causes collagen deposition [54]. This causes the transplanted cells to separate from the recipient, thus hindering the regeneration process. Extrahepatic HT, such as in the spleen and peritoneum (Fig. 1), may be considered a solution to this issue. Seven patients from India received peritoneal infusions of foetal liver cells from 26–34-week-old unborn babies. The results revealed that HT patients had a 10% higher overall survival rate than controls [55]. Birir et al. described five patients with ALF who underwent intrasplenic HT [56]. At 48 h post-transplantation, three of the five patients showed significant improvements in encephalopathy scores, blood ammonia levels, prothrombin time, and overall survival after HT. In other studies, sodium alginate microencapsulation has been used to encapsulate hepatocytes in microspheres, protecting them against the body's immunological response and enabling molecular exchange [31]. Of course, HT also has adverse effects in specific clinical situations. During hepatocyte infusion in the portal vein, intrapleural, or peritoneal, some patients developed deadly mesenteric vein thrombosis [30] and non-lethal splenic vein thrombosis [57]. This shows that there is still room for improvement in hepatocyte delivery.

Acute-on-chronic liver failure (ACLF) is characterized by acute decompensation (AD), organ failure, and high short-term mortality [58, 59]. The development of ACLF frequently occurs due to an unanticipated occurrence, such as bacterial infection, acute alcohol-associated hepatitis, drug-induced hepatitis and viral hepatitis, with a 28-day death rate of 30%. Depending on the grade, regular treatment enables only 16–51% of patients to reverse ACLF, leaving a sizable percentage of patients with ACLF stable or progressing [60]. In a clinical experiment by Wang et al. on patients with ACLF, one patient was bridged to OLT, three restored liver function, and another three died after intrasplenic injection of 4.2–6.0 × 10^10^ hepatocytes [61].

Overall, the application of HT has shown great therapeutic potential for end-stage liver disease. However, this approach has limitations. One of the main challenges is the limited availability and engraftment efficiency of hepatocytes, as well as manufacturing difficulties. These obstacles include difficulties in isolating high-quality cells from donor livers, mechanistic limitations in cryopreserving liver cells while maintaining viability, low levels of engraftment and proliferation in transplanted liver cells, and issues related to cell delivery. It has been shown that long-term proliferation of human hepatocytes (lc-ProliHH) can induce activation of dedifferentiation-associated inflammatory factors (DAIF), thus inducing increased macrophage activation. Blockage of the innate immune response by dexamethasone improved engraftment and repopulation [62]. The strategy of coculturing hepatocytes with IL-6, epidermal growth factor (EGF) and hepatocyte growth factor (HGF) can promote long-term expansion of primary hepatocytes (> 30 passages in ~ 150 days with theoretical expansion of ~ 10^35^ times) and repopulate livers [63]. Recent advances in liver/cholangiocyte organoids have provided a promising solution to address these challenges. Organoids often have higher viability and efficiency in engraftment and proliferation in vivo [64]. Moreover, the delivery issues associated with HT may result from alternative strategies such as intrasplenic cell infusion and peritoneal delivery (Table 1).

**Table 1:** Clinical indication and application in cell therapy

Cell type	Clinical indication	Clinical application	References
Hepatocyte	IEM	Yes	[33–35, 41]
	Crigler–Najjar syndrome type I	Yes	[36]
	ALF	Yes	[48–50, 52, 55, 56, 190]
	ACLF	Yes	[60, 61]
PSC	uPA immunodeficient	No	[87]
Macrophage	Liver fibrosis	Yes	[146, 147]
MSC	Liver fibrosis, chronic liver disease, ACLF	Yes	[117–119]
Haematopoietic progenitor cell	Liver fibrosis	Some clinical reports and small number of randomized trials	[172–174]

*IEM* inborn errors of metabolism, *ALF* acute liver failure, *ACLF* acute-on-chronic liver failure, *PSC* pluripotent stem cell, *uPA* urokinase-type plasminogen activator

#### Foetal liver progenitor cells

In vitro, foetal liver progenitor cells (FLPs) may differentiate into hepatocytes (Fig. 1). It has been shown that foetal hepatocytes from humans and other primates may proliferate and mature in the livers of immunodeficient mice, repopulating 10% of the organ [65, 66]. Furthermore, it has been shown that FLPs can differentiate into functional human endothelial cells and that no tumour development was observed within nine months after transplantation [67]. Additionally, it was demonstrated that liver fibrosis was reduced in an animal model that received transplants of human FLPs and that the majority of the human hepatocytes still present in the mouse livers were from proliferation rather than the original transplant [68]. In an immunodeficient animal model, FLPs have a poorer proliferative ability than adult hepatocytes, despite results showing that FLPs can differentiate into hepatocytes [68, 69]. It is possible that the liver could not provide adequate signals to promote the proliferation and engraftment of FLPs. The issues of inefficient engraftment remain a significant challenge. Some researchers have shown that direct intrahepatic injection of cells within a hyaluronan matrix may significantly improve engraftment [64]. Furthermore, the development and use of FLPs for treating liver disease have also been constrained by ethical debate over the acquisition of foetal tissue.

#### Pluripotent stem cells

Pluripotent stem cells (PSCs), which need to be differentiated into hepatocytes before cell transplantation, are regarded as an alternative cell source for HT (Fig. 1) [70]. To distinguish between embryonic stem cells (ESCs) and induced pluripotent stem cells (iPSCs), several techniques have been developed [71–75]. ESCs are derived from pluripotent stem cells that form in the blastocyst cluster of cells in humans 4–5 days after fertilization. Because ESCs have been researched for a long time, some protocols employ cell development signals to promote the differentiation of ESCs into hepatocyte-like cells (HLCs). Bone morphogenetic protein (BMP) and fibroblast growth factor (FGF) promote hepatocyte development, whereas activin A and WNT3 signalling favour endoderm differentiation [71, 76, 77]. Despite many attempts, the phenotype of HLCs derived from ESCs fails to match that of adult hepatocytes [78]. In addition, immune rejection after ESC transplantation and ethical aspects of ESC acquisition are inevitable issues. In 2013, many research groups used nuclear transplantation to effectively create ESCs from the somatic cells of patients, although the process is still technically challenging [79].

Through the induction of adult fibroblasts, Takahashi's team created iPSCs in 2007 [80]. In addition to human fibroblasts, other cells may be reprogrammed into iPSCs, including keratinocytes, lymphocytes, endothelial colony-forming cells, and mesenchymal stem cells [81–84]. iPSCs are comparable to ESCs in terms of pluripotency, infinite proliferative ability, and plasticity. The benefits of iPSCs for hepatocyte differentiation and organoid culture in vitro have been shown in several studies [75, 81, 85]. Most earlier investigations have shown that HTs and hepatocytes produced from iPSCs have similar transplant efficacy [86]. Nevertheless, research by Carpentier's team showed that by employing iPSCs for implantation in urokinase-type plasminogen activator (uPA) immunodeficient mice, 15% liver regeneration (LR) could be attained [87]. Furthermore, cell transplantation using HLCs differentiated from iPSCs is also associated with a risk of carcinogenesis resulting from genetic mutations. Merkle et al. found that PSCs accumulated single nucleotide variants (SNVs) in cancer-related genes, including the tumour suppressor gene TP53 [88]. Moreover, poor engraftment and immune-mediated loss are regarded as crucial factors [89]. In an attempt to overcome these challenges, a previous study has shown that supplementing the reprogramming media with antioxidants is attributed to the reduction of genomic aberrations in iPSCs [90]. Some studies have used activin A, dimethyl sulfoxide, hepatocyte growth factor, oncostatin M, and dexamethasone to induce hepatocyte maturation [91]. These strategies provide solutions for the engraftment and repopulation of iPSCs. Clinical translation will be substantially accelerated by creating iPSCs with minimal immunogenicity and improved differentiation ability. In conclusion, many modifications are still needed for application of iPSCs in clinical translation.

#### Organoids

The application of organoids has increasingly aroused researchers’ interest. “Organoid” is defined as a three-dimensional (3D) structure that is grown from stem cells or adult cells. Compared with two-dimensional (2D) cell lines, organoids have the advantages of organ structure and genetic stability. Appropriate organoids are considered an important source of available cell therapy [92]. Previous studies have successfully constructed liver organoids from Lgr5^+^ cells and iPSCs that can be applied to treat liver failure in mice [93–95]. Takebe et al. reported that after transplantation, liver organoids can reconstruct functional blood vessels and save mice from drug-induced liver damage [96]. Forbes et al. rescued bile duct structure and function in a mouse model of biliary disease with organoids constructed from purified human biliary epithelial cells (hBECs) [24]. A study from the University of Cambridge found that cholangiocyte organoids can repair bile duct injury after transplantation in the human liver [23].

In summary, organoids show great promise in cell transplantation. Future research could apply liver/cholangiocyte organoids to treat end-stage liver disease and improve the quality of expanded criteria donors in expanded liver donor pools.

### Remodelling the hepatic microenvironment

#### Anti-inflammatory and immunomodulatory effects

**Fig. 2:**
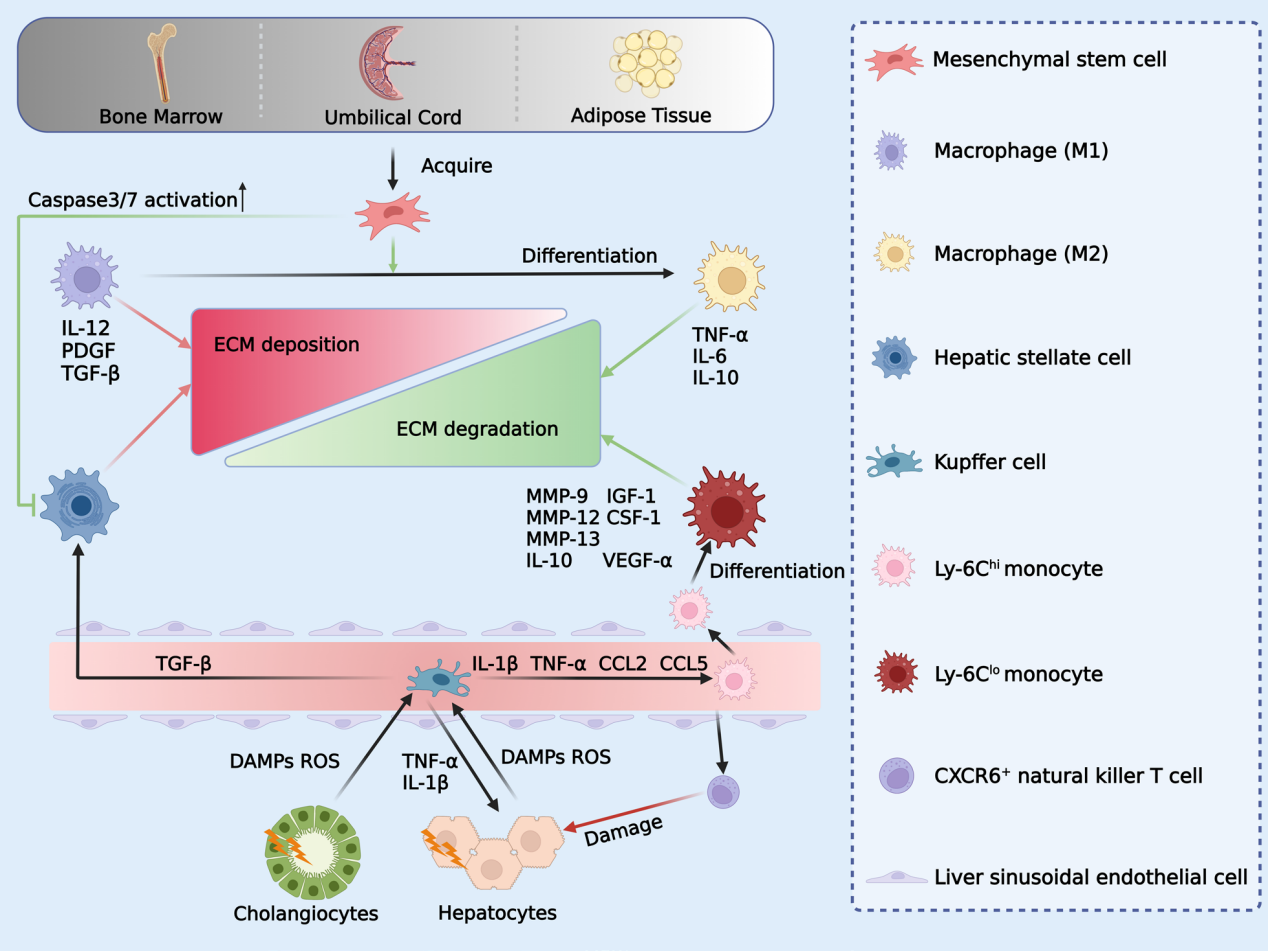
Mesenchymal stem cell and macrophage reduce ECM deposition in the liver. MSCs, which are acquired from bone marrow, umbilical cord, and adipose tissue, can reduce ECM deposition by promoting the polariton of macrophages from M1 to M2 and inhibiting HSCs. Macrophage M1 secretes IL-12, PDGF, and TGF-β to exacerbate ECM deposition. In contrast, macrophages M2 secrete TNF-α, IL-6, and IL-10 to accelerate ECM degradation. Meanwhile, damaged hepatocytes and cholangiocytes secrete DAMP and ROS to activate Kupffer cells in the hepatic blood sinusoids. Activated Kupffer cells could recruit Ly-6C^hi^ monocytes by secreting IL-1, TNF-α, CCL2, and CCL5 and activate HSCs by secreting TGF-β. In contrast, Ly-6C^hi^ monocytes recruit CXCR6^+^ natural killer T cells to damage hepatocytes. Meanwhile, Ly-6C^hi^ monocytes further differentiate into Ly-6C^lo^ monocytes, which can accelerate ECM degradation by secreting MMP-9, MMP-12, MMP-13, IL-10, IGF-1, VEGF-α, and CSF-1

In addition to replacing host hepatocytes, transplanted cells promote LR by remodelling the liver microenvironment. One of the important factors is the immunomodulatory effect of transplanted cells. Mesenchymal stem cells (MSCs) are often employed in the clinic due to their simplicity in isolation, growth, and preservation. MSCs were first isolated in bone marrow [97] and can also be obtained from umbilical cords and adipose tissue (Fig. 2). Nevertheless, MSCs vary in their characteristics depending on the origin of cells and tissues as well as culture conditions [98]. MSCs are in vitro progenitor cells that exhibit plastic adhesion capabilities and a fibroblast-like appearance. MSCs express specific cell surface markers such as CD105, CD73, and CD90 [99]. Studies on MSCs have shown that they can effectively improve immune response [100]. MSCs were discovered in one investigation to increase CCL18, which in turn promoted monocyte survival [101]. Furthermore, by interacting with dendritic cells and decreasing their expression of inflammatory molecules such as IL12, TNF-α, and IFN-γ while increasing their secretion of IL-10 (Fig. 2), MSCs can differentiate antigen-presenting cells towards monocytes, which may lead to an increase in the number of regulatory T cells [102]. Furthermore, MSCs are able to suppress the proliferation and pro-inflammatory cytokine secretion of immune cells through the secretion of anti-inflammatory factors. MSCs improve the anti-inflammatory effects of macrophages by secreting prostaglandin E2 (PGE2), stimulated gene/protein 6, and indoleamine 2,3-dioxygenase (IDO) [103]. Pro-inflammatory cytokines, such as IL-1β and IL-6, were also inhibited by MSCs [104]. Moreover, MSCs inhibit antibody production and secretion and the proliferation of activated B lymphocytes in an IDO-dependent manner [105, 106]. In summary, the anti-inflammatory and immunomodulatory effects of MSCs can be exploited to promote liver regeneration.

Liver sinusoidal endothelial cells (LSECs) constitute the vascular wall of the hepatic sinusoids and are considered key regulators of LR. Loss of LSECs exacerbates inflammation in liver disease [107]. Fang et al. found that LESCs could improve NASH by alleviating inflammation [108]. The underlying cause may be a decrease in NO secretion. LSEC-derived NO can effectively play an immunomodulatory role in the liver. Targeting LSECs for treatment has been shown to be effective [109]. Therefore, it may be a good choice to use LSECs to remodel the liver microenvironment in liver disease.

#### Anti-fibrosis

Fibrosis is one of the main manifestations of end-stage liver disease. MSCs and macrophages can effectively alleviate liver fibrosis and ECM deposition. The primary functions of MSCs are to release numerous trophic factors to suppress hepatic stellate cell (HSC) activation and ECM formation during LR, thus decreasing fibrosis, apoptosis, and inflammation via immunomodulation of T cells, B cells, and macrophages (Fig. 2). Liver disease and fibrosis are closely connected processes. In liver fibrosis and fibrosis regression, macrophages are crucial [110, 111]. Furthermore, macrophages secreting pro-fibrotic factors, including TGF-β and platelet-derived growth factor (PDGF) (Fig. 2), may worsen liver fibrosis when in the M1 state [110–112]. However, the ability of MSCs to polarize macrophages to the M2 state increases the possibility that they may alter the cytokine profile of activated macrophages biologically and reduce fibrosis [113, 114]. The antifibrotic ability of MSCs was further validated by coculture with HSCs in vitro and an animal model of liver fibrosis. Wang et al. reported that when MSCs and HSCs were cocultured, the expression of liver fibrosis markers was considerably decreased [115]. By boosting caspase3/7 activity (Fig. 2), another work by Lin et al. showed that MSCs may prevent the proliferation of activated HSCs and promote their death [116]. Certain clinical trials have confirmed the therapeutic benefits of MSC transplantation in patients with chronic liver disease [117–119]. According to a randomized controlled trial from Lin et al., peripheral infusion of allogeneic bone marrow-derived MSCs was safe and practical for patients with HBV-associated ACLF. By enhancing liver function and lowering the risk of serious infections, this treatment significantly increased 24-week survival [119]. Additionally, it has been shown that MSCs administered by hepatic arterial infusion considerably decreased liver fibrosis in patients and resulted in a notable improvement in Child–Pugh scores [117]. Nevertheless, relative to one transplant, liver fibrosis did not deteriorate after two MSC transplants. For these reasons, MSCs are considered a potential cell source for cell therapy.

Hepatic macrophages are one of the critical cells in the pathophysiology of chronic liver damage and have recently been highlighted as possible antifibrosis targets [120]. Macrophages undergo substantial phenotypic and functional changes after tissue damage and are essential for the initiation, maintenance, and resolution phases of tissue recovery. By changing their phenotype in response to cues from the liver microenvironment, hepatic macrophages may perform several tasks [121]. The typical categorization of macrophages as either "pro-inflammatory" type M1 or "pro-repair" type M2 does not accurately represent how they operate [122]. According to rodent liver damage models, circulating monocytes are mainly categorized as Ly-6C low (Ly-6C^lo^) monocytes and Ly-6C high (Ly-6C^hi^) monocytes (Fig. 2) [123]. The former display patrolling behaviour and express more clearance receptors in the liver, while the latter express "inflammatory" chemokine receptors such as CCR2, pattern recognition receptors, and cytokines [124–128]. The origin of hepatic macrophage subpopulations has a significant impact on how well they function in liver disease [123]. Ly-6C^hi^ monocytes are mainly derived from bone marrow [129], and Ly-6C^lo^ monocytes are mainly derived from the spleen [130]. Kupffer cells (KCs), mainly located in the blood sinusoids of the liver, act as the body's first line of defence against pathogens by effectively identifying and eliminating blood-borne germs, particularly gram-positive germs [131].

Kupffer cells (KCs) are rapidly lost during the damage period after liver injury [132, 133]. Reactive oxygen species (ROS) and damage-associated molecular patterns (DAMPs), such as high mobility group Box 1 (HMGB1), mitochondrial DNA (mtDNA), and ATP, are released by injured hepatocytes or cholangiocytes, and these molecules activate resident KCs on the luminal side of the hepatic sinusoidal endothelium (Fig. 2). After that, KCs quickly release pro-inflammatory cytokines and chemokines such as IL-1β, TNF-α, CCL2, and CCL5, which stimulate hepatocytes to secrete protective or apoptotic signalling pathways and recruit Ly-6C^hi^ monocytes. Ly-6C^hi^ monocytes then boost liver damage by recruiting CXCR6^+^ natural killer T (NKT) cells (Fig. 2) [134, 135]. However, there is evidence that KCs may activate HSCs through a paracrine mechanism and encourage their transdifferentiation into myofibroblasts, worsening liver fibrosis [136, 137]. Macrophages, as one of the primary producers of matrix metalloproteinases (MMPs), may breakdown various ECM proteins, but specific matrix metalloproteinases can also accelerate liver fibrosis [138]. The antifibrotic, pro-ECM degradation and pro-wound healing functions of macrophages are also regulated. For instance, MMPs released by macrophages may accelerate fibrosis regression. Homologous mature macrophages injected into a CCl4-treated animal model raised the levels of the antifibrotic cytokine IL-10 and the regional growth factors IGF-1, VEGF, and CSF-1 by delivering MMP-13 and MMP-9 to the liver scar to prevent the progression of liver scars [139]. In a mouse model of ALF, Kupffer cells recruit Ly-6C^hi^ monocytes to the damaged area and display pro-inflammatory (TNF-α, IL-1, IL-6, CCL2, and CCL5) signals. Subsequently, they directly activate HSCs in a TGF-β-dependent manner while displaying pro-fibrotic (IL-13) phenotypes [137, 140–143]. Ly-6C^lo^ monocytes, on the other hand, have antifibrotic properties (Fig. 2) [138]. In a mouse model of liver fibrosis, CCL2 inhibitors prevented Ly-6C^hi^ monocytes from entering the liver, thus indirectly increasing Ly-6C^lo^ monocytes, and liver fibrosis was shown to significantly decrease [139]. In another study, the conversion of Ly-6C^hi^ to Ly-6C^lo^ by injecting liposomes sped up liver fibrosis recovery [143]. These studies show that while distinct phenotypes of macrophages may carry out opposing tasks, they mostly contribute to fibrosis regression in the case of liver fibrosis (Fig. 2).

Given the critical role of recruited monocytes, specifically Ly-6C^lo^, in promoting the regression of liver fibrosis, the use of exogenously differentiated macrophages in vitro for treating liver illness may be a promising strategy. By injecting bone marrow-derived macrophages (BMDMs) into a mouse model of CCL4-induced liver fibrosis, researchers were able to drastically lower ECM deposition, the number of myofibroblasts (activated HSCs), and MMP-9 levels [139]. Nevertheless, individuals with liver disease, portal hypertension, and soft coagulation may not be candidates for infusion. According to later research, the delivery of primary human monocyte-derived macrophages (MDM) into the spleen of mice with liver fibrosis also has an antifibrotic ability [144]. Qin et al. discovered that the infusion of active macrophages into animals with liver fibrosis via the tail vein could efficiently breakdown collagen in the liver [145]. Recently, patients with liver cirrhosis have been investigated for response to autologous macrophage infusion treatment. Forbes et al. administered autologous macrophage treatment to nine persons with cirrhosis and an MELD score of 10 to 16 (ISRCTN 10,368,050). Each group of three subjects received a single peripheral infusion of cells at 10^7^, 10^8^, or up to 10^9^ [146, 147]. Within a year, all individuals were still alive and transplant-free, meeting the study's primary goal of safety and viability. This work provides a theoretical foundation for cell treatment for cirrhosis and other fibrotic disorders.

In conclusion, MSCs and macrophages are critical to the regeneration of the liver and may serve as a source of cell therapy.

#### Tissue repair and regeneration

Another function of cell therapy is to promote tissue repair and regeneration. Haematopoietic progenitor cells play a major role in this process. Bone marrow (BM) is an alternate source of hepatocytes in the context of liver damage [148–151]. Previous research has shown that BM can generate a range of adult stem cells that express biomarkers for non-haematopoietic progenitor cells [152–154]. Although they may produce hepatocytes under tissue stress, BM cells have little impact on parenchymal replenishment in liver damage [27, 155–158]. The current hypothesis holds that the therapeutic improvement after haematopoietic progenitor cell therapy for damaged liver is primarily mediated by stimulating endogenous progenitor cells through paracrine signalling between the donor and host cells, which provides cytokines and growth factors [158–160]. Studies have shown that following partial hepatectomy in mice and humans, haematopoietic progenitor cells may reduce IL-1-mediated inflammation and boost liver regeneration in a CD39-dependent manner [161]. CD34 and CD133 are frequent markers for collecting haematopoietic progenitor cells [162, 163]. However, human CD34 cells have been shown to have little activity after implantation [164]. According to follow-up research, this may be connected to the activation-dependent expression of CD34, which may have clone-forming properties in multilineage progenitor cells [165, 166]. The metabolic marker aldehyde dehydrogenase (ALDH), which is linked to increased stem cell activity in vivo [165, 166], is found in early immature cells. Currently, a popular strategy is to extract haematopoietic progenitor cells based on vigorous ALDH activity in conjunction with markers such as CD34 or CD133 [168, 169]. Granulocyte colony-stimulating factor (G-CSF), a haematopoietic growth factor, promotes the mobilization of haematopoietic progenitor cells to peripheral circulation [170]. In acute and chronic liver damage models, G-CSF-induced proliferation of haematopoietic progenitor cells has been demonstrated to aid liver regeneration [171, 172]. In recent clinical research, patients with alcoholic cirrhosis who had haematopoietic progenitor cell transplantation achieved long-lasting therapeutic results [172, 173]. E. Yannaki et al. demonstrated that G-CSF hastened recovery and enhanced survival in a model of acute liver damage. In a large randomized controlled clinical study, the combination of G-CSF with the infusion of autologous haematopoietic progenitor cells via the portal vein dramatically boosted survival and liver function (as measured by the Child–Pugh score and liver biochemistry) [174]. However, individuals with liver cirrhosis who received G-CSF or an infusion of G-CSF with autologous CD133 + cells did not have appreciable effectiveness [175]. The diversity of these clinical studies may result from various infusion protocols, infusion techniques, and doses.

LSECs can activate intracellular pathways, including the Notch1 signalling pathway, activation of the transcription factor KLF2, and expression of CD44, vascular cell adhesion molecule 1 (VCAM-1), c-myc and c-jun [176–178]. These molecules have been shown to play a crucial role in LR. Meanwhile, LSECs secrete HGF, Wnt2, NO, IL-6 and TNF-α to promote LR [179, 180]. HGF stimulates hepatocyte proliferation through c-Met [181]. LR failed in a mouse model in which HGF was specifically knocked out in LSECs [182]. Id1 knockout mice have reduced HGF and Wnt2 expression, but the injection of allogeneic LSECs can restore hepatic angiogenesis [180]. LESCs also recruit monocytes to stimulate LR. In CD11b knockout mice, cellular crosstalk between monocytes and LESCs was disrupted, resulting in decreased angiogenesis and survival [183]. In conclusion, LSECs promote liver regeneration by releasing angiocrine factors. Transplanting LSECs to promote LR is a promising therapeutic strategy.

## Optimizing the operation of cell therapy

### Cell delivery

**Fig. 3:**
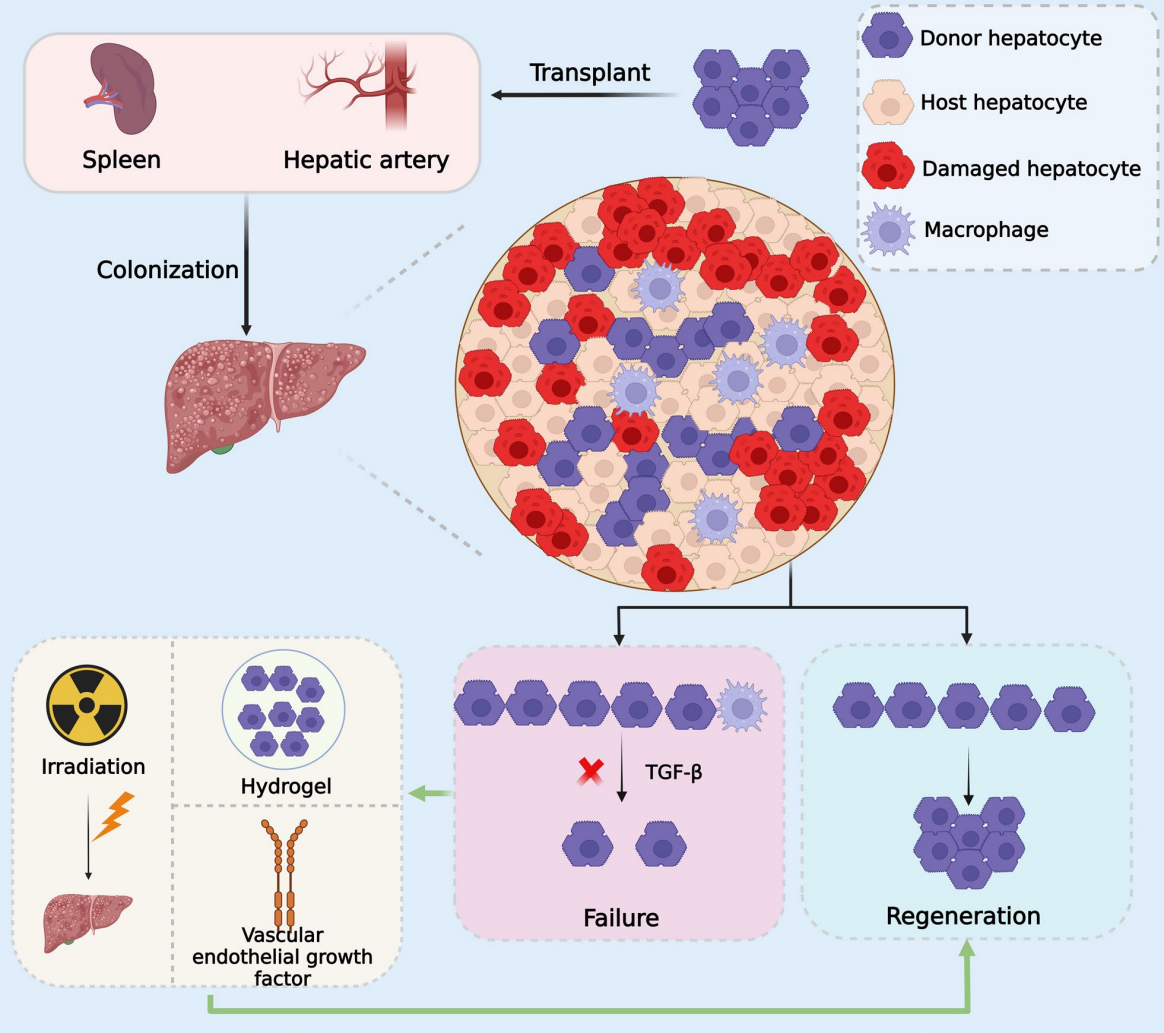
Optimization of hepatocyte transplantation. Hepatocyte transplantation through the hepatic artery or the spleen may result in better colonization. And hepatocytes may be rejected by macrophages after reaching the liver. Macrophages phagocytose donor hepatocytes and secrete TGF-β to induce senescence. In this case, macrophages can avoid phagocytosis by encasing the donor hepatocytes in a hydrogel. On the other hand, the proliferative advantage of donor hepatocytes can be enhanced by irradiating the liver or injecting vascular epithelial growth factors

The liver is highly vascularized; therefore, transplanted cells may be administered by several different pathways, of which the portal vein or hepatic artery are the most common (Fig. 3). The transplanted cells should be delivered to the hepatic sinusoids, where they need to subsequently integrate with the liver parenchyma [184]. The most frequent clinical technique is cell infusion via the portal vein. Three options are available for adults: an intrahepatic splenic vein tributary puncture, an intrahepatic portal vein tributary puncture, and an intrahepatic portal shunt via the jugular vein through the hepatic venous system [185–187]. For newborns, access is via umbilical vein cannulation. In older children, laparoscopic or minimum dissection surgery or an incisional method may be used to place a central venous cannula into the portal vein stent. Depending on the size of the cell, hepatocytes cross-sinusoidal veins, temporarily occluding the periportal vascular area, and normal blood flow is subsequently restored via vascular permeability [49, 188]. Nevertheless, all of these treatment options have a risk of bleeding, particularly for patients with liver illness who often have portal hypertension, collateral circulation formation, and coagulation disorders. The transplanted cells may also reach the pulmonary capillaries via the hepatic veins and produce thrombi there, causing pulmonary infarction. The high portal pressure in individuals with portal hypertension makes it difficult for cells to reach the hepatic sinusoids. Within 24 h, macrophages eliminated all remaining hepatocytes from the portal veins (Fig. 3). Therefore, there are two possible solutions for managing this issue: (1) continuous Doppler ultrasonography monitoring to ensure that the portal pressure does not exceed 12 mmHg [89] and (2) hepatic artery administration of transplanted cells (Fig. 3). In contrast to the portal vein approach, the hepatic artery is often employed as a conduit for internal radiation treatment and arterial chemoembolization and may serve as a more effective way to deliver cells [189]. Other research has suggested alternative strategies, such as intrasplenic cell infusion for cirrhosis patients [52] and peritoneal delivery methods for ALF patients [55, 190]. The advantage of these strategies is that they permit functional liver recovery while preventing hepatic immune rejection.

### Cell recovery and proliferation

The activity and proliferation capacity of donor cells after transplantation play a major role in the effectiveness of cell therapy. Hepatocyte preservation has always been a serious issue. Cryopreservation significantly harms hepatocytes, reducing ATP generation and downregulating integrin-β1 and E-cadherin [191–193]. Hepatocyte acquisition and preservation procedures have considerably improved in recent years, and the activity of rewarmed hepatocytes is comparable to that of fresh hepatocytes [193, 194]. The techniques developed at the University of Wisconsin in the late 1980s are still used in most modern preservation techniques for donor organs and suspended cells [193, 194]. Many laboratories are also beginning to accept newer hepatocyte preservation media, such as Hypothermosol-FRS (HTS-FRS) and Institut Georges Lopez 1 (IGL-1), due to their better quality and lower cost [197–199]. Meanwhile, hepatocyte activity and function have been improved using apoptosis inhibitors and cystathionine inhibitors [200, 201].

Through the blood sinusoids, the transplanted cells enter the liver and attach to the liver parenchyma. It takes 1–5 days for transplanted cells to bind to recipient hepatocytes, and a gap junction, as well as a bile duct network formed by the two types of cells, can be seen in rats [202, 203]. During this process, HSCs are activated, while the implanted hepatocytes express the genes of the host hepatocytes at their location and undergo proliferative activity [204, 205]. Compared to the spleen and peritoneum, the liver exhibits much higher levels of proliferation [206]. This implies that the liver is a better candidate for the transplant location. Another crucial element in the effectiveness of cell therapy is intentionally controlling how tightly transplanted cells adhere to the recipient’s liver. The growth of transplanted cells in rats may be aided by pretreatment techniques based on the production of liver damage [18]. These techniques include boosting the release of advantageous compounds such as vascular endothelial growth factor (VEGF) (Fig. 3) from HSCs and rupturing the endothelial barrier that separates the parenchyma and hepatic blood sinusoids [207–210]. VEGF may also be externally supplied. Additionally, by causing liver damage, irradiation of the LSECs and a portion of the liver lobe (Fig. 3) may also improve the competitive advantage of transplanted cell proliferation [43, 211]. Immune rejection issues are avoided, and the effectiveness of transplantation is increased by coating the transplanted cells with hydrogel in material rich in growth factors and matrix proteins (Fig. 3) [212].

## Conclusions and perspectives

As the demand for liver transplantation grows worldwide, more research is required to bridge the gap between donor livers and waiting list patients, as well as enhance the long-term prognosis of liver transplant recipients. Over the last several decades, cell therapy has significantly advanced, proving its effectiveness and safety. Before it can be utilized in clinical trials, a number of challenges must be resolved, including isolating high-quality cells from donor livers, enhancing cell implantation to reduce immune reactions, and improving engraftment and long-term outcomes. Additionally, stem cells or cells with stem cell qualities may be employed to address the challenges associated with hepatocyte acquisition. HLCs derived from iPSCs and organoids of various cell origins (iPSCs, progenitor cells, cholangiocytes) provide sufficient cell sources for cell therapy. Furthermore, iPSCs and organoids always have higher viability and efficiency in engraftment in vivo. Organoids with genetic stability and highly expandable properties could be a promising strategy for end-stage liver disease. Different cells in the liver have a role in replacing and remodelling the liver microenvironment; thus, combining various cell treatments may be the future tendency of cell therapy. The most significant advantage of cell therapy is a high degree of flexibility in selecting the best cells and transplantation sites for treatment catering to the patient’s needs.

## Data Availability

Not applicable.

## Abbreviation

NAFLD: Non-alcoholic fatty liver disease
NASH: Non-alcoholic steatohepatitis
OLT: Orthotopic liver transplantation
BEC: Bile duct epithelial cell
hBEC: Human bile duct epithelial cell
PSC: Pluripotent stem cell
MSC: Mesenchymal stem cell
LR: Liver regeneration
HSC: Hepatic stellate cell
ECM: Extracellular matrix
MMPs: Matrix metalloproteinases
IEM: Inborn errors of metabolism
HT: Hepatocyte transplantation
ALF: Acute liver failure
TGF-β: Transforming growth factor-β
ACLF: Acute-on-chronic liver failure
AD: Acute decompensation
FLP: Foetal liver progenitor cell
ESC: Embryonic stem cell
iPSC: Induced pluripotent stem cell
HLC: Hepatocyte-like cell
BMP: Bone morphogenetic protein
FGF: Fibroblast growth factor
uPA: Urokinase-type plasminogen activator
SNV: Single nucleotide variants
Ly-6C^lo^: Ly-6C low
Ly-6C^hi^: Ly-6C high
KC: Kupffer cell
ROS: Reactive oxygen species
DAMP: Damage-associated molecular patterns
HMGB1: High mobility group box 1
mtDNA: Mitochondrial DNA
LSEC: Liver sinusoidal endothelial cell
NKT: Natural killer T
BMDM: Bone marrow-derived macrophage
MDM: Monocyte-derived macrophage
BM: Bone marrow
ALDH: Aldehyde dehydrogenase
G-CSF: Granulocyte colony-stimulating factor
HTS-FRS: Hypothermosol-FRS
IGL-1: Institut Georges Lopez 1
VEGF: Vascular endothelial growth factor
PDGF: Platelet-derived growth factor
MDM: Monocyte-derived macrophages
VCAM-1: Vascular cell adhesion molecule 1
HGF: Hepatocyte growth factor
hBEC: Human biliary epithelial cell
lc-ProliHH: Long-term proliferation human hepatocytes
DAIF: Dedifferentiation-associated inflammatory factors
EGF: Epidermal growth factor
PGE2: Prostaglandin E2
IDO: Indoleamine 2,3-dioxygenase
